# Therapeutic effect of Yinhuapinggan granules mediated through the intestinal flora in mice infected with the H1N1 influenza virus

**DOI:** 10.3389/fmicb.2024.1394304

**Published:** 2024-04-29

**Authors:** Can Yang, Jing Chen, Huifen Zhou, Di Zeng, Haitong Wan, Jiehong Yang

**Affiliations:** ^1^School of Basic Medical Sciences, Zhejiang Chinese Medicine University, Hangzhou, Zhejiang, China; ^2^School of Life Sciences, Zhejiang Chinese Medicine University, Hangzhou, Zhejiang, China

**Keywords:** Yinhuapinggan granule, influenza, influenza A virus subtype H1N1, intestinal flora, molecular docking

## Abstract

**Objective:**

In this study, we examined the therapeutic effects of Yinhuapinggan granules (YHPGs) in influenza-infected mice. We also examined how YHPGs affect the composition of the intestinal flora and associated metabolites.

**Methods:**

We used the nasal drip method to administer the influenza A virus (IAV) H1N1 to ICR mice. Following successful model construction, the mice were injected with 0.9% sterile saline and low (5.5 g/kg), medium (11 g/kg), and high (22 g/kg) doses of YHPGs. The pathological changes in the lungs and intestines were evaluated by gavage for 5 consecutive days. Detection of sIgA, IL-6, TNF-α, INF-γ, and TGF-β cytokine levels in serum by enzyme-linked immunosorbent assay. Real-time fluorescence quantitative polymerase chain reaction and Western blot were used to measure the mRNA and protein expression of the tight junction proteins claudin-1, occludin, and zonula occludens-1 (ZO-1) in the colon. To assess the influence of YHPGs on the intestinal microbiota, feces were obtained from the mice for 16s rRNA sequencing, and short-chain fatty acids (SCFAs) were measured in the feces.

**Results:**

By reducing the production of pro-inflammatory cytokines and increasing the relative expression of claudin-1, occludin, and ZO-1 in colon tissues, YHPGs had a protective effect in tissues from the lungs and colon. When YHPGs were administered to mice with IAV infection, the relative abundance of *Lactobacillus*, *Coprobacillus*, *Akkermansia*, *Prevotella*, *Oscillospira*, and *Ruminococcus* increased, whereas the relative abundance of *Desulfovibrio* decreased.

**Conclusion:**

The therapeutic mechanism of YHPGs against IAV infection in mice may be underpinned by modulation of the structural composition of colonic bacteria and regulation of SCFA production.

## 1 Introduction

The influenza virus is the primary cause of influenza, an acute respiratory disease that is primarily spread through the air (Basu et al., 2014). There are four types of influenza virus: A, B, C, and D, with the type A virus being the primary cause of pandemic influenza (Nelson and Holmes, 2007). Inflammation, fever, headache, cough, sore throat, and muscle aches are symptoms associated with influenza virus infection (Ampomah and Lim, 2020). Neuraminidase inhibitors are currently the primary treatment for influenza in clinical practice. However, new influenza virus strains with increased drug resistance are emerging as a result of the extremely changeable nature of the influenza virus (Shie and Fang, 2019). Therefore, domestic and international research has focused on the creation of novel medications with low toxicity, low drug resistance, and high efficacy.

When the influenza virus enters the host, pattern recognition receptors are activated, which triggers the cytokine storm. The cytokine storm involves excessive release of inflammatory mediators and chemokines (Gu et al., 2019). Acute respiratory distress syndrome (Nouveau et al., 2021) and multi-system organ failure (Liuzzo and Patrono, 2021) are just two of the complications resulting from this systemic inflammatory response.

According to Zhu et al. (2018) intestinal damage in mice caused by influenza virus infection involves decreased expression of Zonula occludens-1 (ZO-1), a tight junction protein, and a consequent increase in intestinal permeability. Previous research has shown that antibiotic therapy predisposes mice to high viral replication in the lungs, and that dysregulated intestinal flora in mice results in decreased adaptive immunity, which lowers the body’s ability to clear the influenza virus (Ichinohe et al., 2011). Moreover, Shi et al. (2020) discovered that lung damage in mice infected with the influenza A virus (IAV) may be prevented by altering the balance of T helper 17/regulatory T cells in the gut–lung axis. As a result, altering the gut–lung axis appears to be a promising method to treat influenza.

Yinhuapinggan granules (YHPGs) are an innovative Chinese medicine. YHPGs contain Lonicerae Japonicae Flos, Ephedra Herba, *Amygdalus Communis* Vas, Polygoni Cuspidati Rhizoma Et Radix, Licorice, and Radix Puerariae. YHPGs have been given the national invention patent of YHPG (Patent No. ZL03151188.0) and the certificate of new Chinese medicine (Z20120004) for their effectiveness in eliminating heat, detoxifying and moistening the lungs. Previous research has shown that YHPG has significant antiviral effects in infected mice, in part through inhibition of influenza virus replication and modulation of influenza virus infection-induced apoptosis (Peng et al., 2016). Also our research group found that by limiting influenza virus replication and controlling the incidence of apoptosis brought on by influenza virus infection, YHPG exhibits strong antiviral effects in IFV-infected mice. These findings raise the possibility that YHPG is a promising antiviral drug with future therapeutic application opportunities. However, information on whether YHPGs treat influenza by altering the intestinal flora is lacking.

In this study, we examined how the intestinal flora of mice with IAV-induced pneumonia influences the ability of YHPGs to prevent influenza and the mechanisms underlying the therapeutic effects of YHPGs.

## 2 Materials and methods

### 2.1 Materials

#### 2.1.1 Laboratory animals and viruses

The license number for the production of experimental animals was SYXK (zhe)2021-0001. Seventy male ICR mice weighing 18 ± 2 g were purchased from Hangzhou Medical College. Influenza A/PR/8/34 virus (H1N1 subtype) was donated by the Chinese Academy of Sciences (The Institute of Basic Medical Sciences and Oncology). The virus was adapted to mouse lungs and their tissue culture infective doses (TCID50) were 10^–3.4^. The study was conducted by the guidelines of the Animal Ethics Committee of Zhejiang Province and the ethical number is 2023R0014.

#### 2.1.2 Medicines and reagents

The ingredients in the YHPG formula were obtained from the Chinese herbal pharmacy of the Hall of Famous Traditional Chinese Medicine at Zhejiang University of Traditional Chinese Medicine. They included 10 g Lonicerae Japonicae Flos, 10 g Radix Puerariae, 10 g Polygoni Cuspidati Rhizoma Et Radix, 5 g Ephedra Herba, 5 g *Amygdalus Communis* Vas, and 2.5 g Licorice. The aforementioned herbs were soaked in eight-times as much water for 30 min, decocted for 40 min, and then filtered. The dregs were then added to six-times as much water, decocted for an additional 40 min, and then filtered once more. After combining the two filtrations, the decoction was concentrated to 50 mL, with 1 mL being equal to 0.85 g raw herbs. Shuanghuanglian oral liquid (SHL) was obtained from Henan Fusen Pharmaceutical Co. Ltd. (Lot No. 21042411), and oseltamivir phosphate capsules were obtained from Hoffmann-La Roche (Basel, Switzerland; Lot No. M1301). The bicinchoninic acid (BCA) assay kit was obtained from Biyuntian Biotechnology Company. The SuperScript^™^ III and TRIzol kits were purchased from Thermo Fisher. The Applied Biosystems SYBR^®^ Green PCR Master Mix kit was purchased from Applied Biosystems. Shenggong Bioengineering (Shanghai) Company Limited supplied the real-time fluorescence quantitative polymerase chain reaction (qRT-PCR) primers. Anti-rabbit microbial antibodies against claudin-1 and β-actin were acquired from Abcam, against occludin was purchased from CST, and against ZO-1 was purchased from Thermo Fisher. Secondary goat anti-mouse immunoglobulin G (IgG) (H + L) and goat anti-rabbit IgG (H + L) antibodies were purchased from Thermo Pierce. Mouse Tumor Necrosis Factor alpha (TNF-α) ELISA kit, Mouse gamma interferon (IFN-γ) ELISA kit, Mouse secretory immunoglobulin A (sIgA) ELISA kit, Mouse Interleukin 6 (IL-6) ELISA kit and Mouse transforming growth factor beta (TGF-β) ELISA kit were purchased from Hangzhou Risda Biotechnology Co.

#### 2.1.3 Equipment

The equipment used in this study was as follows: CFX384 multiplex qRT-PCR instrument (Bio-Rad, US); low-temperature high-speed freezing centrifuge (Eppendorf, Germany); ultraviolet spectrophotometer (Beckman, US); protein electrophoresis and transfer system (Bio-Rad); 7890B GC System for gas chromatography (Agilent); 5977B MSD mass spectrometer (Agilent); QT-1 Vortex Tester (Shanghai QITE Analytical Instruments Co. Ltd.).

### 2.2 Methods

#### 2.2.1 Mouse grouping and modeling

Fifty-six mice were randomly and equally assigned to each of the following treatment groups: control, model, oseltamivir, SHL, and YHPG low (YHPG-L) (5.5 g/kg), medium (YHPG-M) (11 g/kg), and high (YHPG-H) (22 g/kg). Mice in all groups, excluding the control group, were anesthetized using a gas anesthesia machine with 20 mL isoflurane. Except for the mice in the control group, others were exposed to 50 μL of 10 × LD50 IAV (H1N1) through intranasal instillation. None of the infected mice died. The dose of YHPGs was 9.1-times the clinical dose used in humans (converted by average body surface area assuming a body mass of 70 kg) according to calculations based on the difference in body weight between humans and mice. The low, medium, and high doses of YHPGs were set at 5.5 g/kg, 11 g/kg, and 22 g/kg, respectively, based on the daily dose of YHPGs supplied to adults, which is equivalent to 85 g of raw YHPGs, and the daily dose of YHPGs administered to mice was 11 g/kg. The above doses were gavaged into the infected mice 24 h after IAV infection, and the treatment group received one gavage every day for 5 days. An equal amount of saline was administered to the control and model groups simultaneously. These regimens were continuously administered throughout the duration of the experiment.

#### 2.2.2 General observation and changes in lung and spleen indices

Each day, the mice were monitored for signs, food and water intake, hair color, and activity. Five days after the final treatment, the mice were sacrificed to collect the lungs and spleen, which were washed in phosphate-buffered saline, dried on filter paper, and weighed. The lung–spleen index was calculated as follows: Lung index = lung weight (g)/body weight (g) × 100%. Splenic index = spleen weight(g)/body weight (g) × 100%.

#### 2.2.3 Pathological changes in the lungs and colon tissues

Fresh lung and colon tissues were fixed with 4% paraformaldehyde, dehydrated, paraffin-embedded, and cut into 4-μm serial sections. The tissues were stained with hematoxylin and eosin (H&E), and the pathological changes in the lung and colon tissues were observed under a light microscope.

#### 2.2.4 qRT-PCR to identify fluctuating viral titer in the lung tissues of IAV virus-infected mice

Bioengineering (Shanghai) Co. designed quantitative PCR primers using Beacon Designer 7.8 and Primer Premier 6.0 software. Table 1 display the primer sequences. To obtain the lung homogenate, a portion of the colon tissue was obtained, ground, and centrifuged at 4000 *g* for 15 min at 4°C. According to the manufacturer’s instructions, total RNA was extracted using Trizol, reverse transcribed into complementary DNA, and subjected to PCR amplification (parameters: 95°C for 2 min, 95°C for 15 s, 62°C for 30 s, 72°C for 30 s, ×40 cycles). The analysis of each sample was performed in triplicate. Analyses of each sample were performed in triplicate, GAPDH was used as the internal reference gene, and the 2^−ΔΔ^Ct method was used.

**Table 1:** The primers for qRT-PCR.

Gene	Forward (5′-3′)	Reverse (5′-3′)
FluA	GACCRATCCTGTCACCTCTGAC	AGGGCATTYTGGACAAAKCGTCTA
GAPDH	GAAGGTCGGTGTGAACGGATTTG	CATGTAGACCATGTAGTTGAGGTCA

#### 2.2.5 ELISA (enzyme-linked immunosorbent assay) to detect serum levels of sIgA, IL-6, TNF-α, INF-γ, TGF-β and other cytokines

To collect blood, use endotoxin- and pyrogen-free tubes, let it clot for 30 min at room temperature, centrifuge it at 1000 × *g* for 10 min, and then carefully separate the serum. Add 50 μL of standard and diluted samples into each of the microtiter wells of the pre-coated enzyme plate. Then, add a detection antibody labeled with horseradish peroxidase to each well and incubate for 60 min at room temperature. Finally, wash the wells and add the chromogenic substrate. Finally, incubate for 15 min away from light. Finally, the color development was terminated and the absorbance at 450 nm was measured within 30 min after the addition of the termination solution (Table 2).

**Table 2:** The primers for qRT-PCR.

Gene	Forward (5′-3′)	Reverse (5′-3′)
Claudin 1	CCTGCCCCAGTGGAAGATTTACT	GTGCTTTGCGAAACGCAGGACAT
Occludin	GCGATCATACCCAGAGTCTTTC	GGTGTCTCTAGGTTACCATTGC
ZO-1	CCATGACTCCTGACGGTTGGTCTT	CGGATCTCCAGGAAGACACTTGT
GAPDH	GAAGGTCGGTGTGAACGGATTTG	CATGTAGACCATGTAGTTGAGGTCA

#### 2.2.6 qRT-PCR to measure the mRNA expression of the tight junction proteins claudin-1, occludin, and ZO-1 in mucosal tissue

To obtain the colon homogenate, a portion of the colon tissue was obtained, ground, and centrifuged at 4000 *g* for 15 min at 4°C. Experiments are performed as in 2.4 (Table 2).

#### 2.2.7 Western blot to measure the relative expression of claudin-1, occludin, and ZO-1 in mucosal tissues

A portion of mouse colon tissue was harvested and ground by adding RIPA buffer. The sample was centrifuged and the supernatant was taken as the total protein solution. The total protein extraction kit (containing Protease Inhibitor Cocktail) was used to extract the total protein. Equal amounts of protein (25 μg) were taken after measuring the protein concentration using the BCA method, and the protein was separated by 10% sodium dodecyl sulfate-polyacrylamide gel electrophoresis. The gel was transferred to a polyvinylidene fluoride (PVDF) membrane, which was blocked with 5% skimmed milk for 1 h at room temperature. The PVDF membranes were then incubated with primary antibodies against occludin (1:1000), claudin-1 (1:2000), ZO-1 (1:500), and β-actin (1:10000) at 4°C overnight.

After incubation with horseradish peroxidase-conjugated sheep anti-rabbit and sheep anti-mouse IgG secondary antibodies (1:5000) for 1 h at room temperature, the enhanced chemiluminescence kit was used for color development, and the gray value of each band was measured using ImageJ software. The expression of each target protein was calculated relative to β-actin as the internal reference protein.

#### 2.2.8 High-throughput sequencing of 16s rRNA in enteric bacteria

The V3–V4 regions of 16 s rRNA were sequenced by I lluminaMiSeq sequencing technology. The microbial information was obtained from the fecal samples by Alpha diversity analysis (Chao1 index, Shannon index, Faith_pd index, Simpson index), Beta diversity analysis [non-metric multidimensional scaling (NMDS) analysis, intergroup difference analysis], taxonomic composition analysis, and other analytical methods.

#### 2.2.9 Determination of colony short-chain fatty acid (SCFA) metabolites

The samples were thawed on ice, and 30 mg of each sample was added to a 2 mL glass centrifuge tube. A total of 900 μL 0.5% phosphoric acid was added to resuspend the sample, and the sample was shaken and mixed for 2 min. After centrifugation at 14000 *g* for 10 min, 800 μL supernatant was taken. An equal amount of ethyl acetate was added for extraction, and the sample was shaken and mixed for 22 min, before being centrifuged at 14000 *g* for 10 min. A total of 600 μL upper organic phase was taken, to which 4-methyl valeric acid at a final concentration of 500 μM was added as the internal standard. The sample was mixed well and added into the injection bottle for gas chromatography–mass spectrometry (GC–MS). For GC–MS detection, the injection volume was 1 μL with a shunt ratio of 10:1. The inlet temperature was 250°C, and the program heating was as follows: initial temperature 90°C, warming to 160°C at a rate of 10°C/min, and warming to 240°C at a rate of 40°C/min and maintained for 5 min. The chromatographic peak area and retention duration were extracted using MSD ChemStation software. The SCFA concentration in the samples was estimated after the standard curve was drawn.

### 2.3 Molecular docking technology to detect YHPG targets

First, the active components and targets of YHPG were screened and corrected using the TCMSP and Uniprot databases. The disease targets of influenza were screened in the GeneCards database, the OMIM database, and the Therapeutic Target Database, and the intersecting targets with YHPG were obtained. Cytoscape and the STRING database were used to assess the Core targets. For molecular docking with potential core targets in the PPI network, identify the primary active components from the “active ingredient-potential targets” network.

### 2.4 Statistical analysis

GraphPad Prism 8.0.1 and SPSS 22.0 were used for the statistical analysis. Normally distributed data are reported as the mean ± standard deviation. One-way analysis of variance was used to compare the data among several groups.

## 3 Results

### 3.1 Effects of YHPGs on the general state of mice and on lung and spleen indices

**Figure 1:**
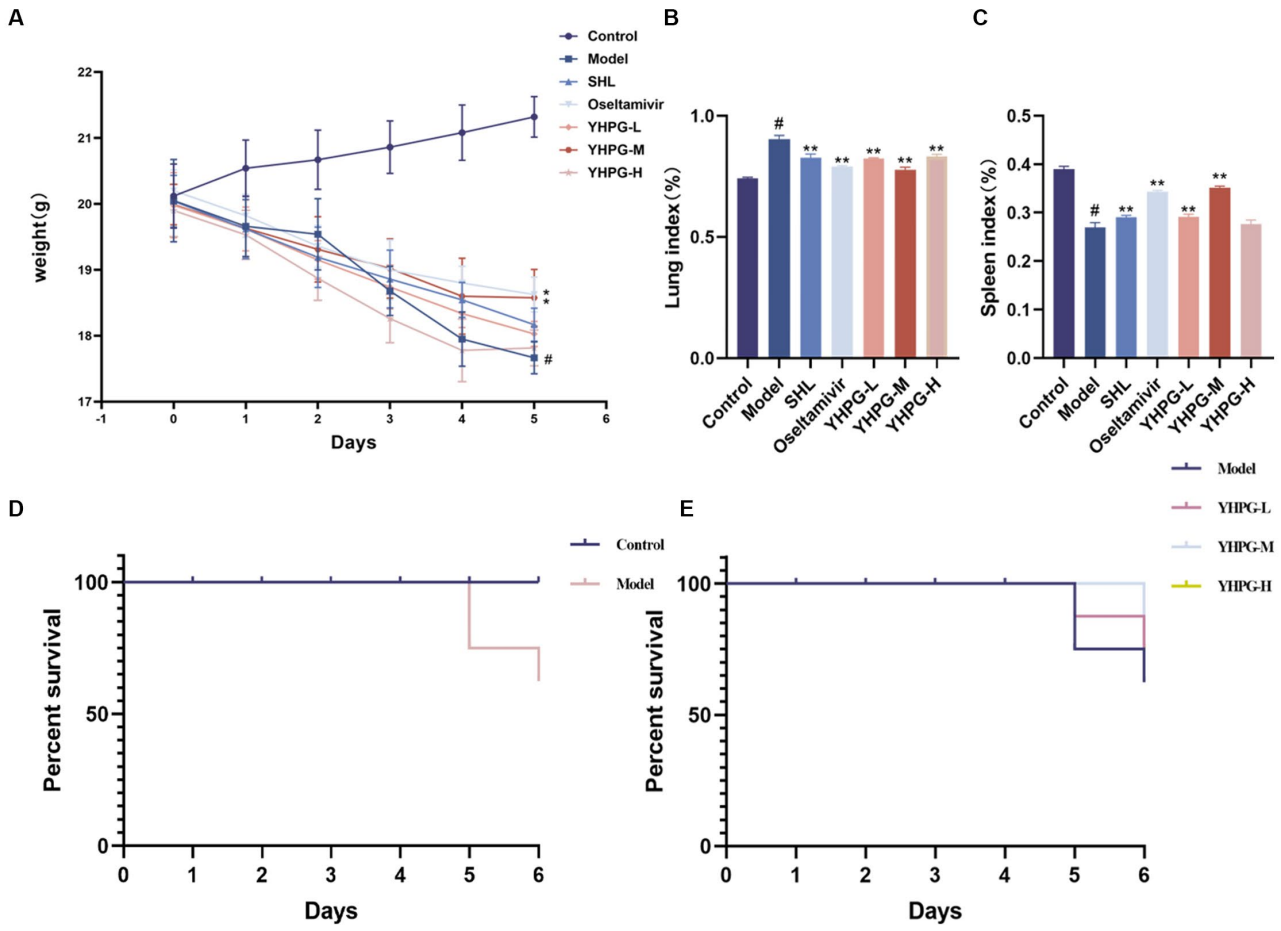
Changes in body weight and lung–spleen index after IAV infection in mice. The mice demonstrated a considerable reduction in body weight and spleen index after 5 days of IAV infection **(A,C)**. The lung index significantly increased in mice after IAV infection **(B)**. The survival rates of mice in each group are shown in panels **(D,E)**. ^#^*p* < 0.05 vs. control group, ^*^p < 0.05 vs. model group, ^**^p < 0.01 vs. model group.

The mice in the control group were psychologically healthy, had lustrous fur, had sensitive behavior, and naturally gained weight. Two days after contracting the IAV, the mice in the model group displayed classic symptoms of influenza, including loss of lustrous fur, a bowed back, a curled body, and shortness of breath. They also gradually lost body weight (*p* < 0.05). Fur color change, shortness of breath, and arched back were significantly less pronounced (*p* < 0.05) in mice in the YHPG-M group than in the model group. The changes in body weight of the mice in each group are shown in Figure 1.

As shown in Figures 1B,C, the lung index of mice in the model group significantly surpassed that of the control group, while the spleen index significantly underperformed the control group (*p* < 0.01). Mice in the YHPG-M group demonstrated a higher spleen index and a lower lung index than mice in the model group (*p* < 0.01).

After the IAV attack, the mice perished on the fourth day, and an autopsy showed that the lung tissue had swollen and become a dark red color. Following the administration of YHPGs, the survival rate of the mice in comparison to the model group did not exhibit a statistically significant variation. Nevertheless, the YHPG intervention caused the mice’s mortality to be postponed. The results are shown in Figures 1D,E.

### 3.2 Effects of YHPGs on the histopathological changes of lung and colon tissues from mice

The alveolar structure of lung tissues from mice in the control group was clear and complete. The alveolar wall was thin, there was no inflammatory secretion in the alveolar lumen, and there was no inflammatory cell infiltration according to the results of H&E staining.

Compared with the control group, mice in the model group demonstrated significantly more inflammatory cell infiltration in the alveolar lumen, a thicker alveolar septum, and vascular congestion. The alveolar septa did not demonstrate significant thickening in the lung tissues from mice treated with YHPGs, and there was less inflammatory cell infiltration. The SHL group demonstrated focal solid areas infiltrated with a large number of inflammatory cells. There were still a few inflammatory cells infiltrating the alveoli in the oseltamivir group.

**Figure 2:**
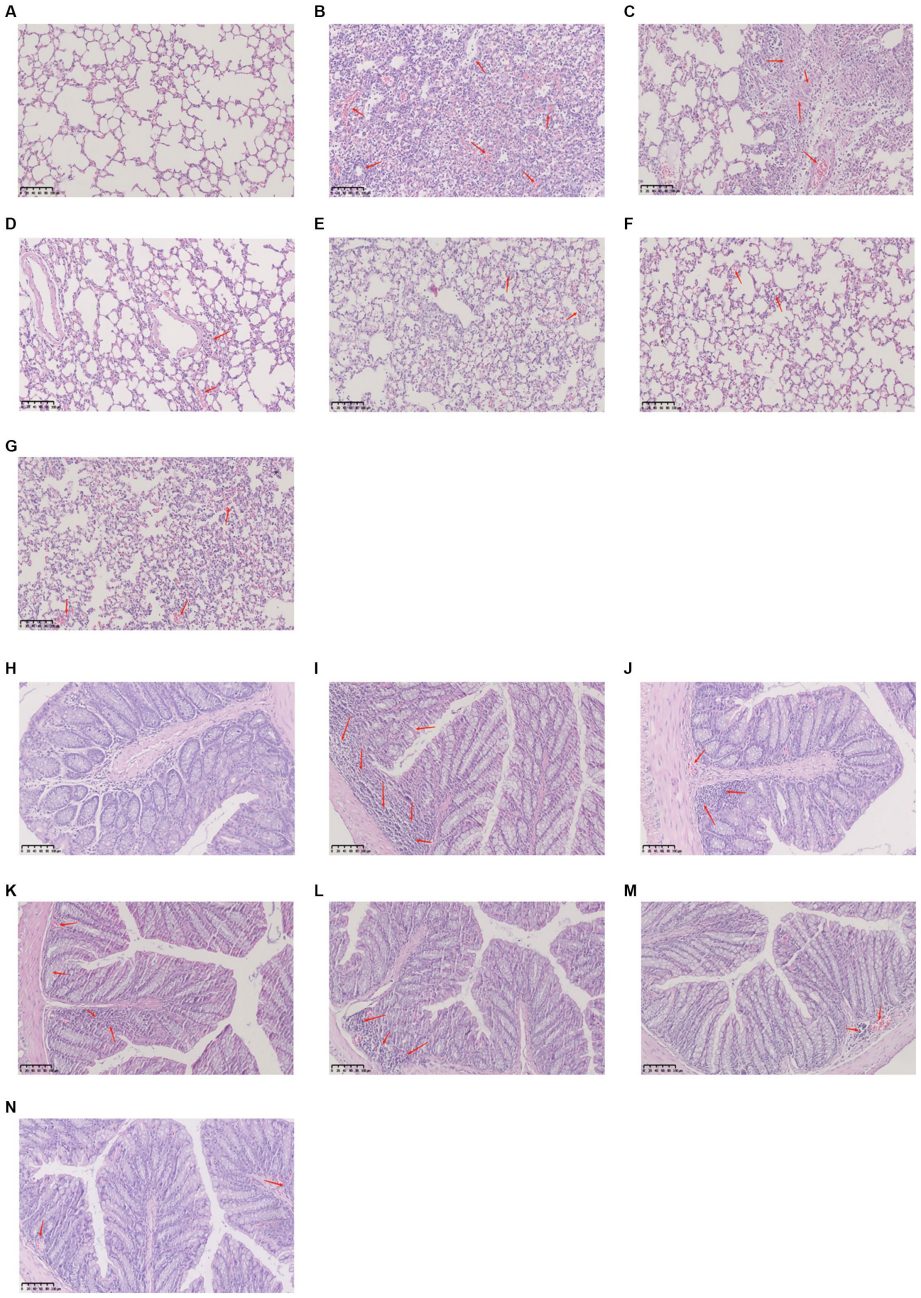
The lungs are shown in pathologic **(A–G)**, while the colon is shown in pathologic images **(H–N)**. The lungs of mice from the control group had no inflammatory cell infiltration and were structurally unharmed **(A)**, whereas the model group demonstrated significant inflammatory cell infiltration and alveolar congestion **(B)**. The SHL group displayed a large number of diseased areas **(C)**, and the lungs of mice in the oseltamivir group and the YHPG group demonstrated minimal inflammatory cell infiltration **(D–G)**. The control group demonstrated a clear colonic structure with neatly arranged glands **(H)**, and the model group demonstrated a disorganized arrangement of cup cells with a large number of infiltrating inflammatory cells **(I)**. The YHPG-L, oseltamivir, and SHL groups only demonstrated minor inflammatory cell infiltration **(J–L)**, and the YHPG-M and YHPG-H groups demonstrated no obvious inflammatory cell infiltration **(M,N)**.

The colonic mucosal epithelial tissue of mice in the control group was unaltered, the glands were arranged in an orderly fashion, the structure was unobscured, there were no ulcers or erosions, and there were no overt abnormalities in the mucosa and submucosal muscularis propria. The intestinal epithelial tissue of mice in the model group was only partially complete compared with the control group, with a disordered arrangement of cup-shaped cells and significant inflammatory cell infiltration. Although the oseltamivir group, the SHL group and the YPHG-L group performed better than the model group, there were still some inflammatory cell infiltration. Neither the YHPG-M nor the YHPG-H group had any overt inflammatory cell infiltration (Figure 2). This implies that YHPGs significantly improve the lungs and intestines of mice with IAV infection.

### 3.3 Effect of YHPGs on viral titer variations in the lung tissue of mice

**Figure 3:**
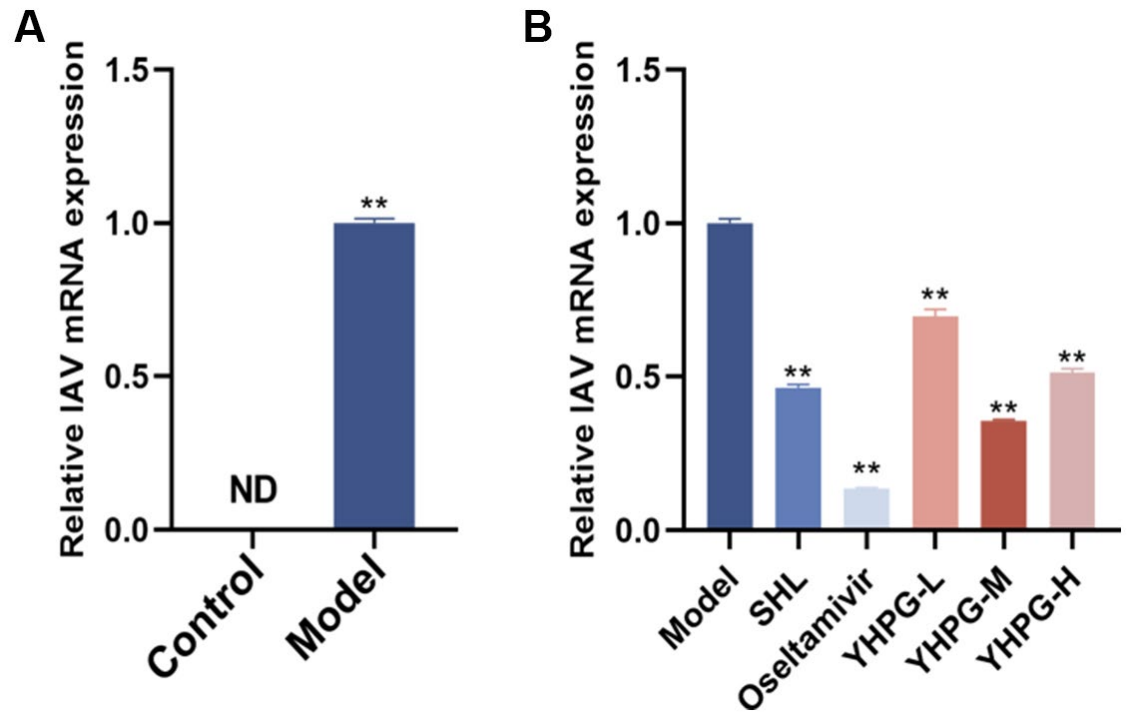
Variations in the viral titer in IAV mice’s lung tissue. The model group showed a significantly higher IAV viral titer in the lung tissues **(A)**. Both the positive drugs group and all YHPG dose groups showed significant decreases in the viral titer in lung tissues **(B)**. ^**^p < 0.01 vs. control group, ^*^p < 0.05 vs. model group, ^**^p < 0.01 vs. model group.

The control mice had no virus, according to the qRT-PCR data. The model group displayed a significantly higher IAV viral titer (*p* < 0.01) than the control group. Compared with the model group, the viral titer was significantly decreased in both the positive drug group and the administered group, in which the middle dose group was the most effective among all the dose groups of YHPGs. The results are shown in Figure 3.

### 3.4 Effect of YHPGs on serum levels of sIgA, IL-6, TNF-α, IFN-γ, TGF-β and other cytokines

**Figure 4:**
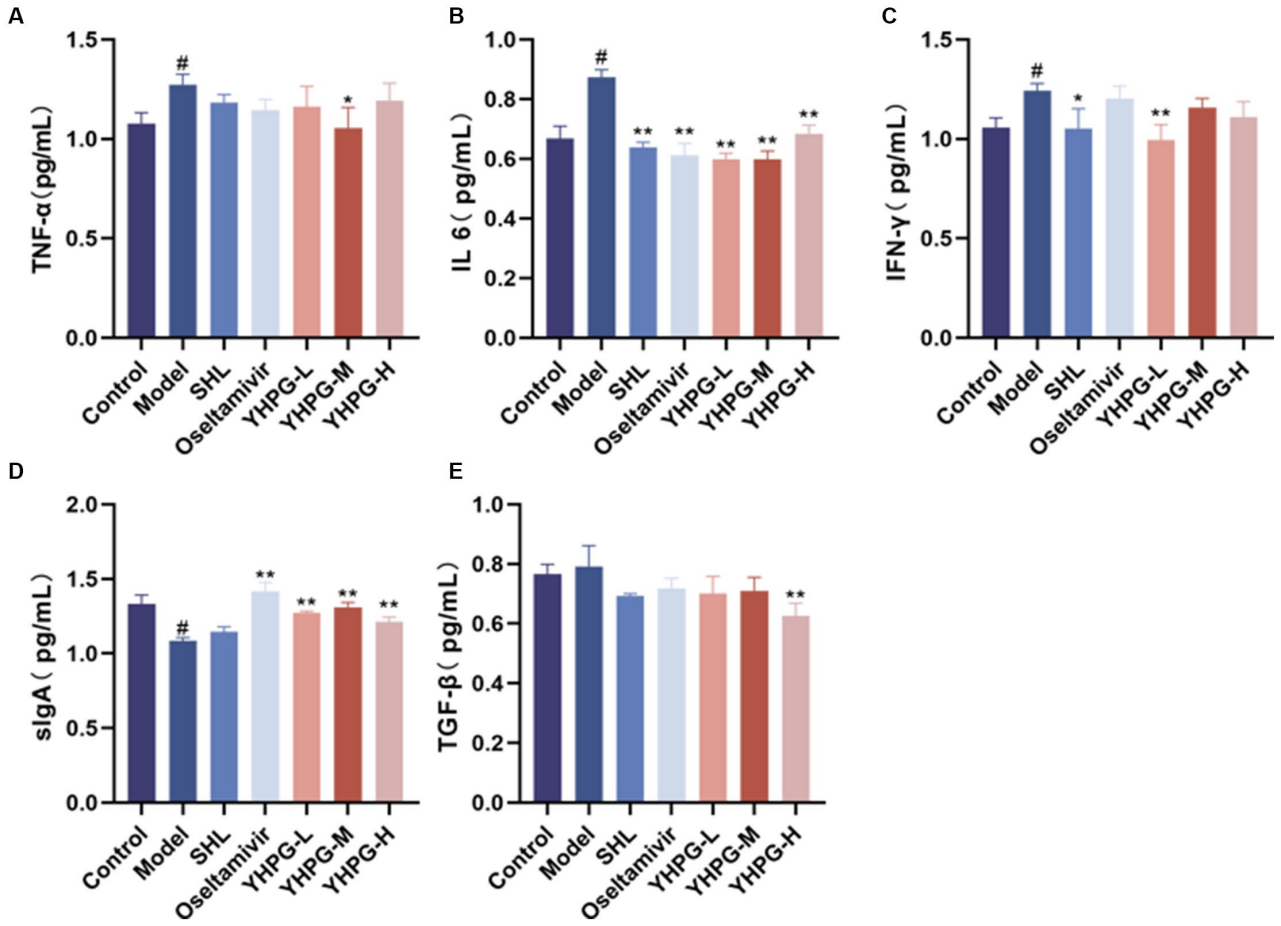
Serum levels of sIgA, IL-6, TNF-α, IFN-γ, TGF-β and other cytokines by ELISA **(A–E)**. The ELISA results showed that the levels of TNF-α and IL-6 were significantly higher and the levels of sIgA was significantly lower in the model group compared with the control group. In these groups, IL-6 and TNF-α levels were significantly decreased in the oseltamivir group and the YHPG-M group, with sIgA levels significantly increased. ^#^*p* < 0.05 vs. control group, ^*^p < 0.05 vs. model group, ^**^p < 0.01 vs. model group.

According to data, TNF-α, IL-6, and IFN-γ levels of the model group were much greater than the control group, whereas sIgA level was significantly lower. The IL-6 level of the SHL group, the oseltamivir group and the YHPG groups had much lower than the model group, while IFN-γ level of the YHPG-L group had significantly lower than the model group. The TNF-α level of the YHPG-M group had lower than the model group. The TGF-β level was significantly greater in the YHPG-H group. The sIgA level was significantly higher in the oseltamivir group and YHPGs groups (Figure 4).

### 3.5 Effect of YHPGs on claudin-1, occludin, and ZO-1 mRNA and protein expression

**Figure 5:**
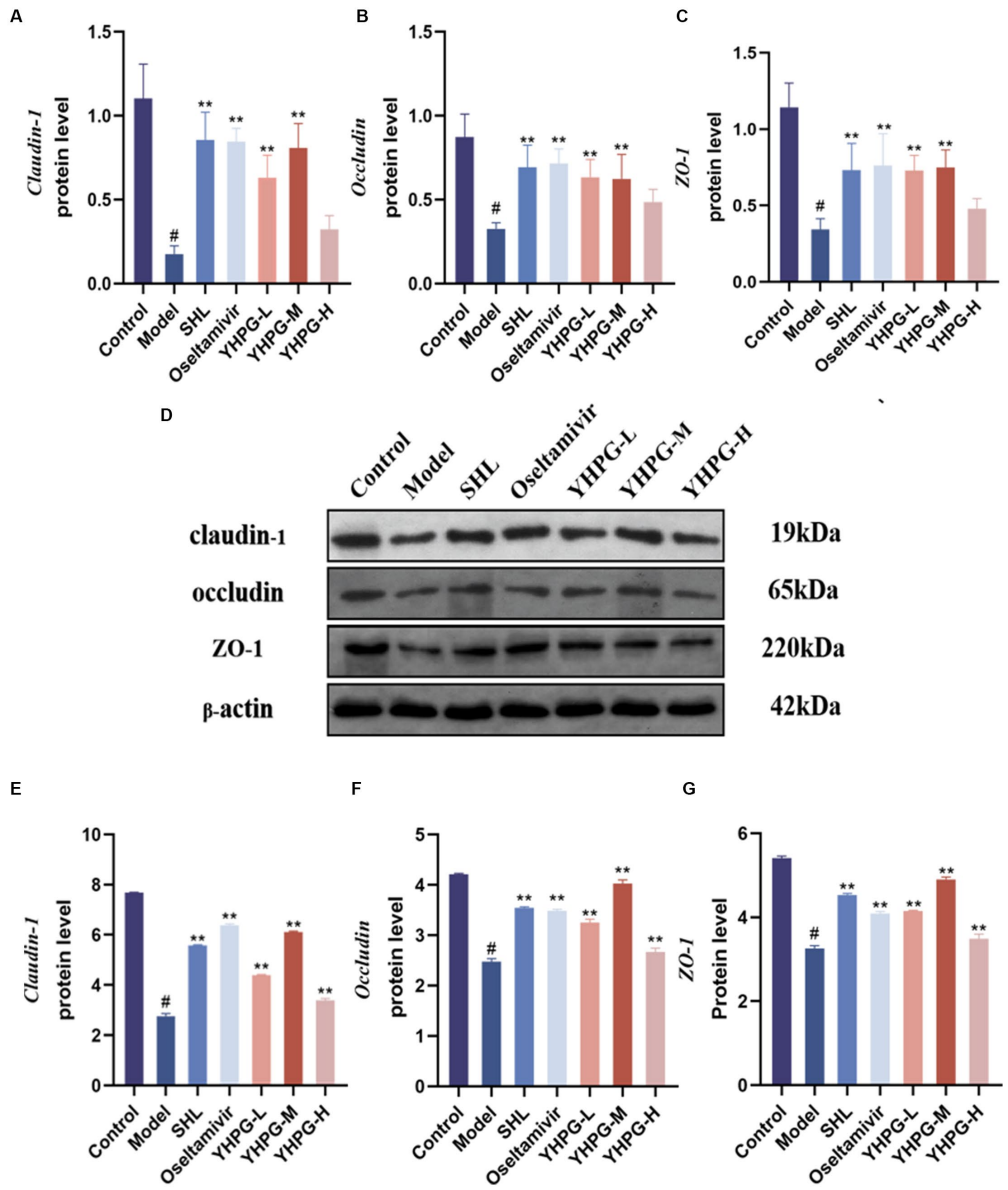
The mRNA expression of claudin-1, occludin, and ZO-1 was measured by qRT-PCR in the colon **(A–C)**. Claudin-1, occludin, and ZO-1 protein expression was measured by Western blot in the colon **(D–G)**. **(D)** Shows a plot of the Western blot result bands from top to bottom for claudin-1, occludin, ZO-1 and the internal reference. Claudin-1, occludin, and ZO-1 mRNA and protein expression were significantly lower in the model group than in the control group, while they were both significantly higher in the drug administration group. ^#^*p* < 0.05 vs. control group, ^**^p < 0.01 vs. model group.

The mRNA expression of the tight junction proteins claudin-1, occludin, and ZO-1 was significantly lower in the colon tissues of mice from the model group than in the colon tissues of mice from the control group according to qRT-PCR (*p* < 0.05). The mRNA expression of these tight junction components was significantly higher in the colon tissues from the positive group, the YHPG-L group, and the YHPG-M group than in the colon tissues from the model group (*p* < 0.01). The protein expression of claudin-1, occludin, and ZO-1 in the colon tissues from mice in the model group was lower than from mice in the control group (*p* < 0.05) according to Western blot. Claudin-1, occludin, and ZO-1 protein expression was significantly higher in the colon tissues from mice in the YHPG groups and the positive drug group than in colon tissues from the model group (*p* < 0.01) (Figure 5).

### 3.6 Analysis of the composition of the intestinal microflora and differential species analysis at the phylum and genus levels

**Figure 6:**
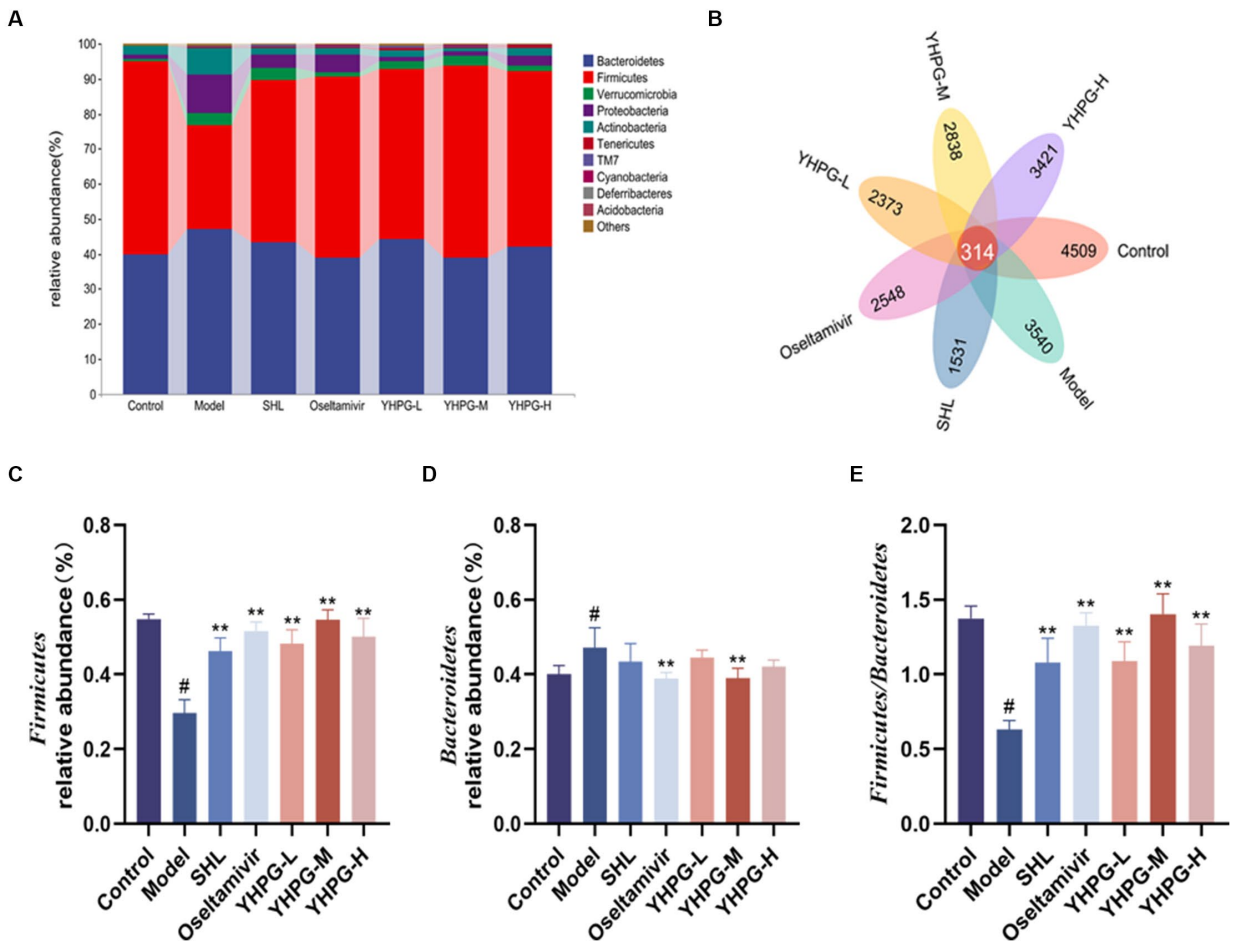
The intestinal flora of mice was analyzed at the phylum level. *Bacteroidetes* and *Firmicutes* were the two phyla with the highest abundance **(A)**. The Venn diagram shows that a total of 314 OTUs were shared between the groups of mice, with a higher similarity of OTUs between the YHPG-M group and the control group **(B)**. Compared with the control group, the relative abundance of *Firmicutes* in the model group was significantly lower **(C)**, while the relative abundance of *Bacteroidetes* was significantly higher **(D)**. The F/B of the model was significantly decreased **(E)**. Compared with the model group, the relative abundance of *Firmicutes* in the positive drug groups and the three groups of YHPG were significantly higher, and the relative abundance of *Bacteroidetes* in the oseltamivir and YHPG-M groups was significantly lower. The F/B was significantly higher in the positive drug groups and the three groups of YHPG. ^#^*p* < 0.05 vs. control group, ^**^p < 0.01 vs. model group.

In the Venn diagram, the overlap of the seven groups of mice represents the OTUs shared between the groups, which totaled 314, based on the number of OTUs studied at the phylum, order, family, genus, and species classification levels, respectively. There were 872, 761, 633, 973, and 614 overlapping OTUs between the SHL group, the oseltamivir group, the low-middle-high-dose group of YHPGs with the control group, respectively. This result shows that the YHPG-M group had a higher similarity of OTUs with the normal group, as shown in Figure 6B.

Using QIIME software to count the characterization table after deleting singletons and focusing on the structural composition of the mouse flora at the phylum and genus levels, the structural composition of the mouse flora at the phylum and genus levels was examined. The categorical column clustering diagram of the top 10 species in each group at the phylum level is shown in Figure 6A. Ten phyla, including *Bacteroidetes*, *Firmicutes*, *Verrucomicrobia*, *Proteobacteria*, *Actinobacteria*, *Tenericutes*, *Saccharibacteria* (*TM7*), *Cyanobacteria*, *Deferribacteres*, and *Acidobacteria*, accounted for the majority of microbes in the mouse colon. The two phyla with the greatest abundance were *Firmicutes* and *Bacteroidetes*. Compared with the control group, the relative abundance of *Bacteroidetes* was significantly higher (*p* < 0.05), whereas the relative abundance of *Firmicutes* was significantly lower (*p* < 0.05), in the model group. F/B is the ratio of *Firmicutes* to *Bacteroidetes*. The F/B was significantly lower in the control group than in the model group. Compared with the model group, the relative abundance of *Bacteroidetes* was considerably lower in the oseltamivir and YHPG-M groups (*p* < 0.01). In the positive group and the YHPG groups, the relative abundance of *Firmicutes* was significantly increased (*p* < 0.01). The F/B in the positive group and YHPG groups was significantly higher (*p* < 0.01). This indicates that the intestinal flora of the IAV-infected mice was significantly disorganized. YHPGs improved the dysfunctional structure of the flora caused by IAV infection, and the effect was marginally superior to that of the two positive drug groups (Figures 6C–E).

**Figure 7:**
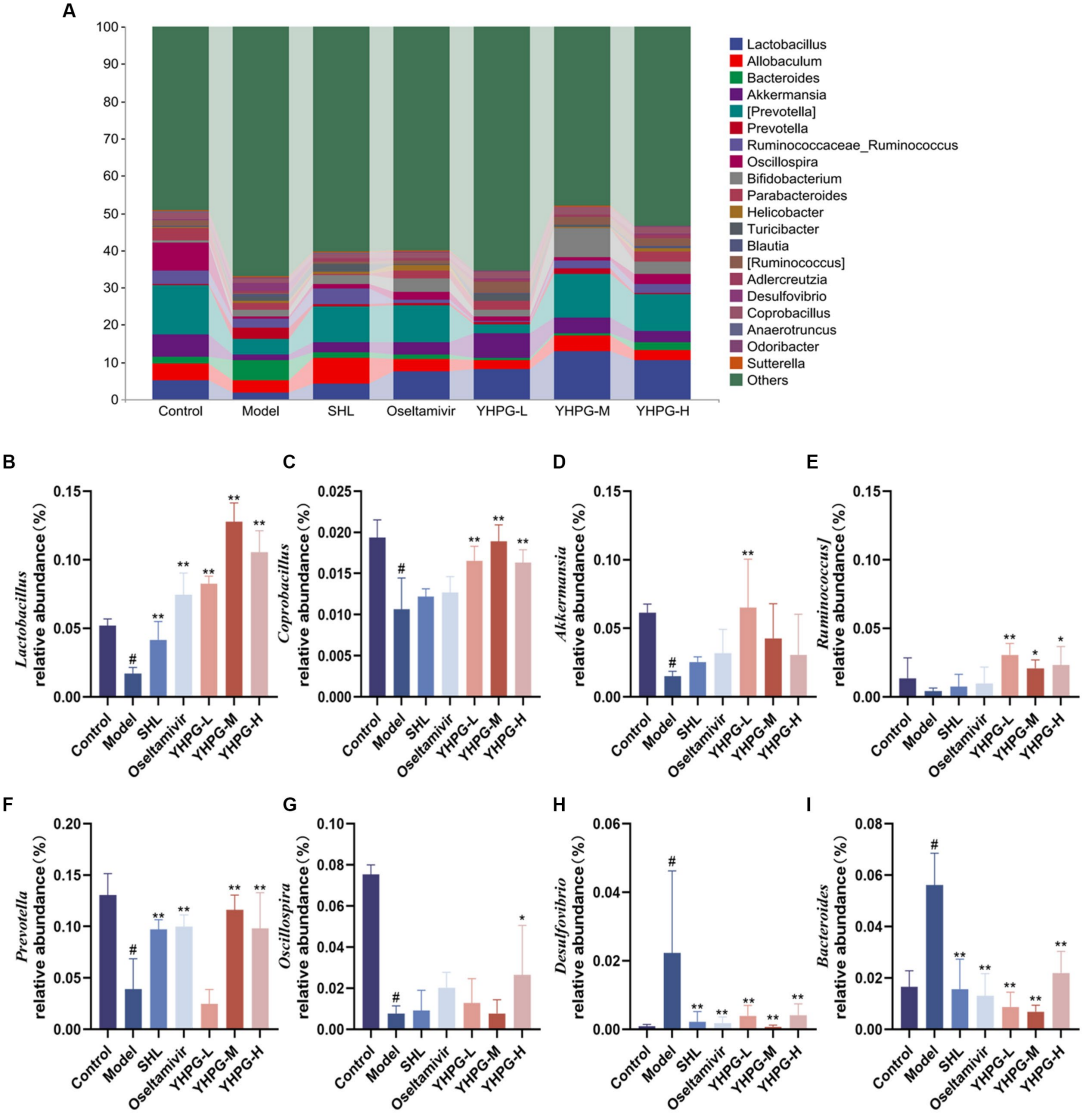
Analysis of the colonic flora at the genus level in mice **(A)**. *Lactobacillus* **(B)**, *Coprobacillus* **(C)**, *Akkermansia* **(D)**, *Ruminococcus* **(E)**, *Prevotell* **(F)**, and *Oscillospira* **(G)** exhibited a significant decrease in relative abundance, while *Allobaculum* **(H)** and *Bacteroides* **(I)** showed a substantial increase in relative abundance, in the model group compared with the control group. The YHPG groups demonstrated a substantial decrease in *Desulfovibrio* and *Bacteroides* abundance compared with the model group, whereas *Lactobacillus*, *Coprobacillus*, *Akkermansi*, *Ruminococcus*, *Prevotel*, and *Oscillospira* increased significantly. ^#^*p* < 0.05 vs. control group, ^*^p < 0.05 vs. model group, ^**^p < 0.01 vs. model group.

The top 20 results for each group at the genus level are displayed in Figure 7A. In the model group, *Lactobacillus* (Figure 7B), *Coprobacillus* (Figure 7C), *Akkermansia* (Figure 7D), *Prevotella* (Figure 7F), and *Oscillospira* (Figure 7G) had relative abundances that were significantly lower than those of the control group (*p* < 0.05). The relative abundance of *Ruminococcus* (Figure 7E) was lower in the model group than in the control group. *Desulfovibrio* (Figure 7H) and *Bacteroides* (Figure 7I) demonstrated a considerable increase in relative abundance (*p* < 0.05). The positive group and each YHPG group demonstrated significantly higher relative abundance of *Lactobacillus* than the model group (*p* < 0.01). Each YHPG group demonstrated a considerably higher relative abundance of *Coprobacillus* and *Ruminococcus* (*p* < 0.05 and *p* < 0.01, respectively), while the YHPG-M group demonstrated a significantly higher relative abundance of *Akkermansia* (*p* < 0.01). *Prevotella* had a substantially higher relative abundance in the positive group, YHPG-M, and YHPG-H groups (*p* < 0.01), while *Oscillospira* had a significantly higher relative abundance in the YHPG-L group (*p* < 0.05). In the SHL group, oseltamivir group and the YHPGs groups, the relative abundance of *Desulfovibrio* was significantly lower (*p* < 0.01). The relative abundance of *Bacteroides* was significantly lower in the positive group and the three YHPG groups (*p* < 0.01).

### 3.7 Alpha diversity analysis and beta diversity analysis indices

**Figure 8:**
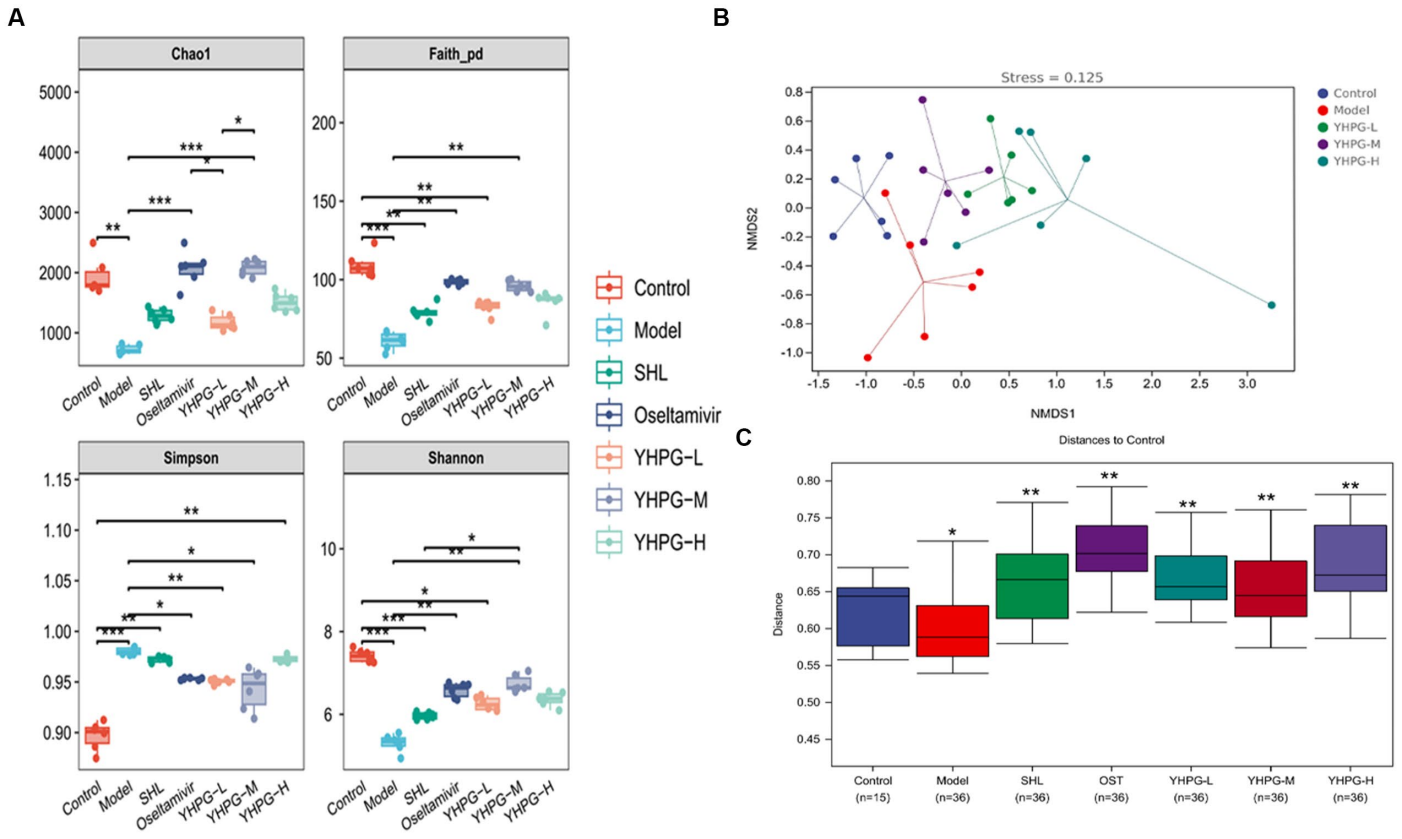
The colon flora of mice was analyzed on an Alpha and Beta scale. In the model group, the Chao1, Shannon, and Faith_pd indices were significantly lower, and the Simpson index was significantly higher than in the control group. The Chao1, Faith_pd, and Shannon indices were considerably higher in the oseltamivir and YHPG-M groups than in the model group, while the Simpson index was significantly lower in the oseltamivir, YHPG-L, and YHPG-M groups. The control group and the model group were differentiated by the Beta analysis **(A–C)**, and the YHPG-M group tended toward the direction of the control group. This implies that YHPGs reduce the difference in the makeup of the gut flora between mice with IAV infection and healthy mice. ^*^p < 0.05, ^**^p < 0.01, ^***^p < 0.001.

Alpha diversity and Beta diversity indices are used by ecologists to characterize the diversity of species inside and within habitats and to assess their overall diversity. The Chao1 index, Shannon index, Faith_pd index, and Simpson index are the key indicators used to assess Alpha diversity. Figure 8A demonstrates that the Chao1, Shannon, and Faith_pd indices in the model group were significantly lower than in the control group (*p* < 0.01), while the Simpson index was much higher (*p* < 0.01). The Chao1, Faith_pd, and Shannon indices were significantly higher than in the model group (*p* < 0.01). Simpson indices were considerably lower in the oseltamivir, YHPG-L, and YHPG-M groups compared to the model group (*p* < 0.05, *p* < 0.01, *p* < 0.05). The findings demonstrate that after infection with the IAV, the richness and diversity of the intestinal flora in mice significantly decreased. YHPGs were able to regulate and balance the dysfunctional intestinal flora to a certain extent and increase the richness and diversity of the intestinal flora in mice.

The findings of the NMDS analysis, which was performed using the unweighted distance algorithm through the R programming language, are displayed in Figure 8B. It is generally accepted that the results of the NMDS analysis are more reliable when the value of stress is <0.2. The model group and the control group were kept apart, and the stress value was <0.2 at 0.125. The data of the YHPG-L and YHPG-M groups were grouped and exhibited a propensity to approach the control group. They were closer to the center of the control group than the model group, with a smaller space between them. The findings of the examination of intergroup differences using the unweighted distance algorithm and NanoSim test are displayed in Figure 8C. The distance between the model group and the control group (*p* < 0.05) and the distance between the administered group and the control group (*p* < 0.01) differed significantly. This suggests that there are differences in gut microbial composition between the control group and the model group. Meanwhile, YHPG can regulate the composition of intestinal microbial species and reduce the difference in the composition of intestinal flora between mice infected with influenza virus and normal mice.

### 3.8 Colony SCFA metabolite content

**Figure 9:**
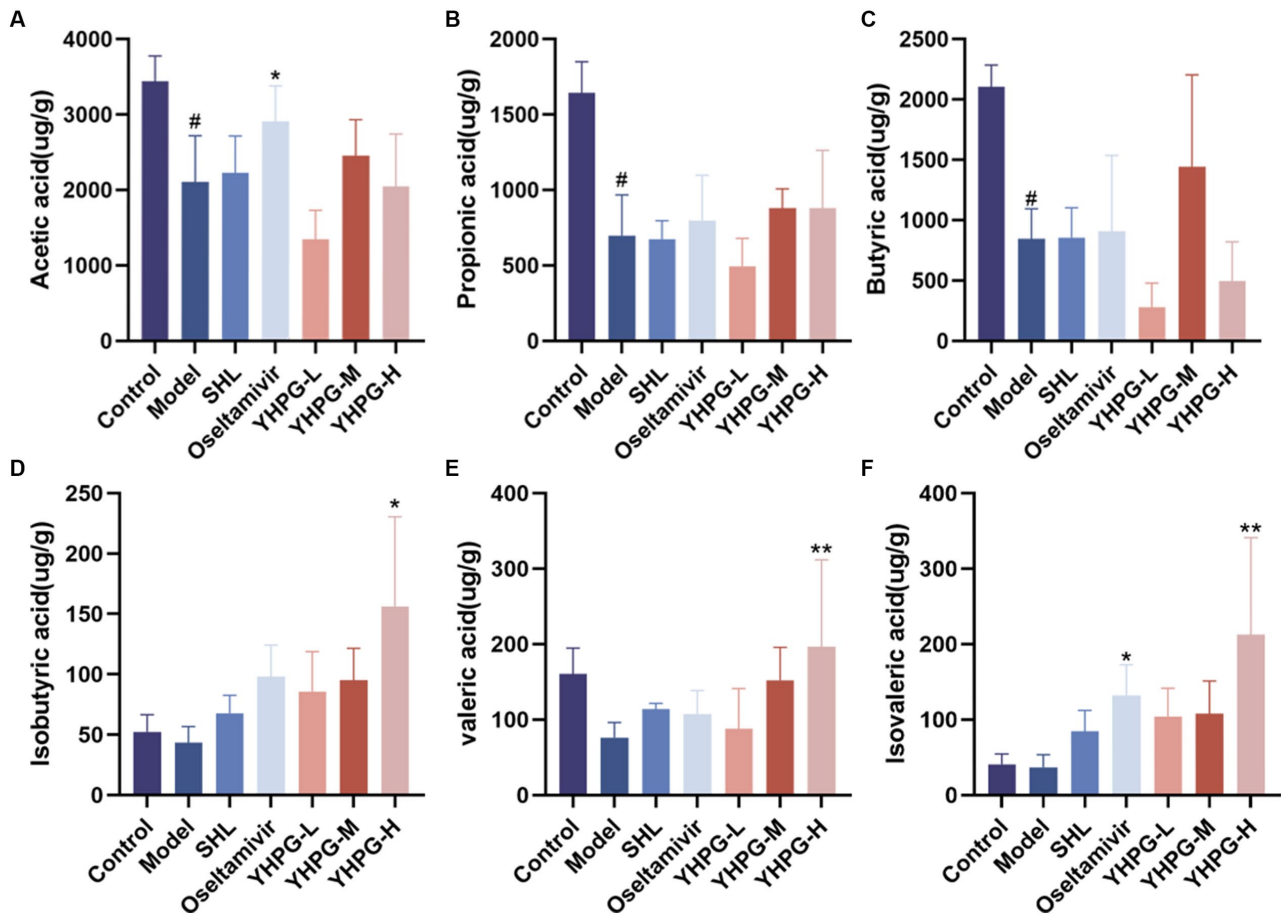
Gas chromatography analysis to identify short-chain fatty acids in mouse feces. Acetic acid **(A)**, propionic acid **(B)**, and butyric acid **(C)** in the feces of mice from the model group were considerably lower than in mice from the control group. In the oseltamivir group, the concentrations of acetic acid and isovaleric acid **(F)** were significantly higher than in the model group, while the concentrations of isobutyric acid **(D)**, valeric acid **(E)**, and isovaleric acid in the YHPG-H group were significantly higher than in the model group. ^#^*p* < 0.05 vs. control group, ^*^p < 0.01 vs. model group, ^**^p < 0.01 vs. model group.

The concentrations of the six SCFAs were measured in the feces of mice from each group, and the differences between the groups are depicted in Figure 9. The quantities of acetic acid, propanoic acid, and butyric acid in the excrement of the model group’s mice were much lower than those of the mice in the control group (*p* < 0.05). The concentrations of isobutyric acid, valeric acid, and isovaleric acid showed a declining trend. Compared to the model group, acetic acid, and isovaleric acid showed a significant increase in the oseltamivir group (*p* < 0.05, *p* < 0.05), and isobutyric acid, valeric acid, and isovaleric acid showed a significant increase in the YHPG-H group (*p* < 0.05, *p* < 0.01, *p* < 0.01).

### 3.9 Molecular docking of YHPG

**Figure 10:**
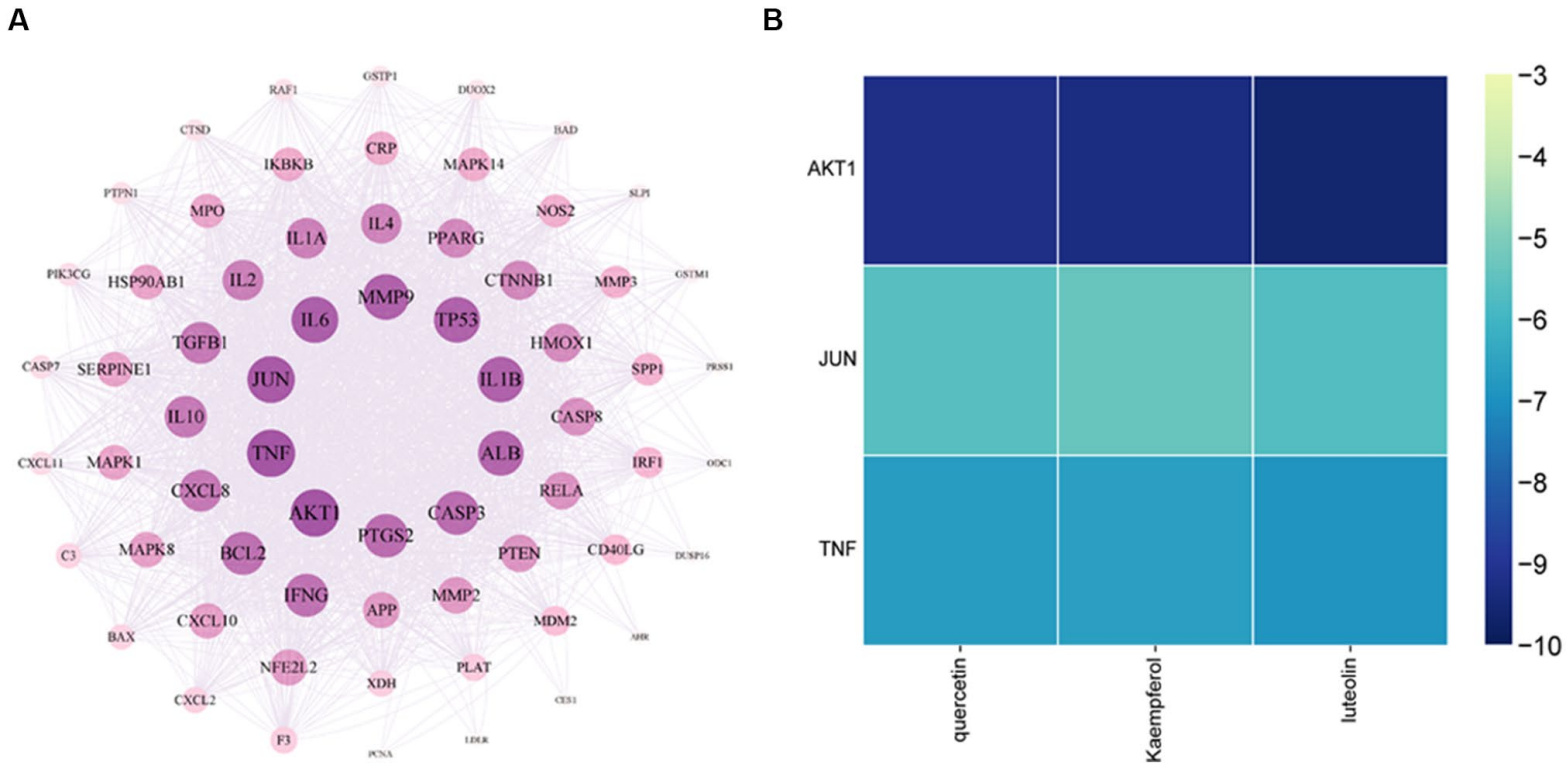
Core target network diagram of YHPG **(A)** and Heat map of quercetin, kaempferol, and luteolin binding to AKT1, JUN, and TNF **(B)**. The degree values of TNF and AKT1 as well as JUN were the top three. AKT1 has the strongest binding capacity and a more stable molecular conformation.

Sixty-seven previously discovered common targets were added to the STRING database for in-depth investigation to investigate the interactions between proteins better. The data was imported into Cytoscape software for additional optimization, as demonstrated in Figure 10A, and the studied protein interaction network diagram, which has 67 nodes and 1,041 edges, was displayed. The results showed that the degree values of TNF and AKT1 as well as JUN were the top three.

**Figure 11:**
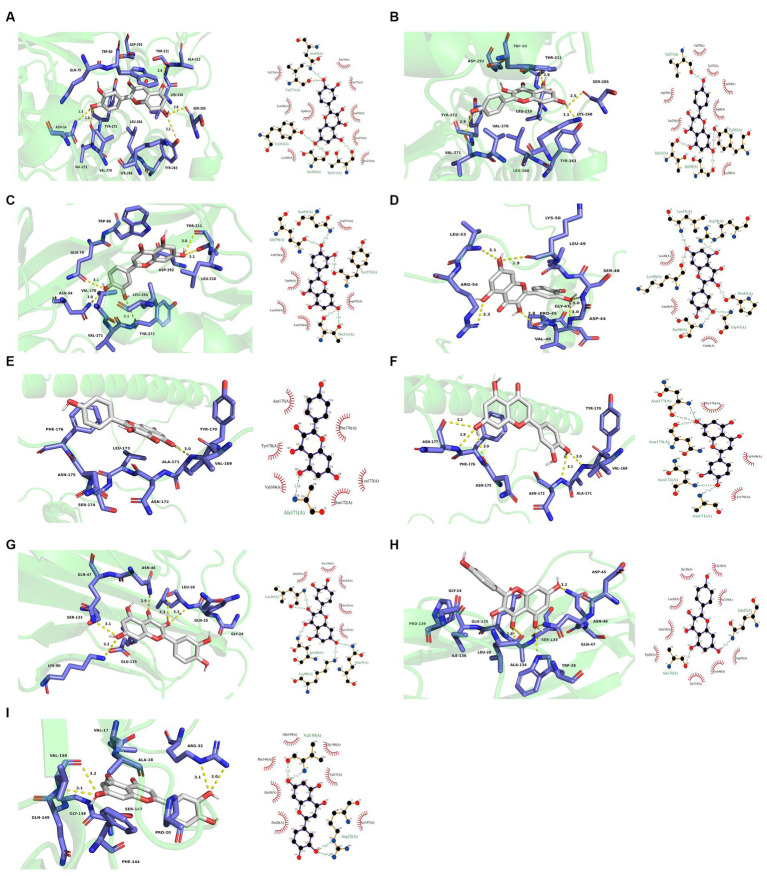
Molecular docking results. **(A)** 3D and 2D plots of AKT1-quercetin butt. **(B)** 3D and 2D images of AKT1-kaempferol butt. **(C)** 3D and 2D images of AKT1-luteolin buttling. **(D)** 3D and 2D images of JUN-quercetin butt. **(E)** 3D and 2D images of JUN-kaempferol docking. **(F)** 3D and 2D images of JUN-luteolin docking. **(G)** 3D and 2D plots of TNF-quercetin butt. **(H)** 3D and 2D images of TNF-kaempferol buttling. **(I)** 3D and 2D images of TNF-luteolin buttling.

AKT1, TNF, and JUN were the molecular docking partners for quercetin, luteolin, and kaempferol (Figure 11). The binding energy of the target and its associated compounds was shown to be negative in the docking data, suggesting that the two had a good binding impact. AKT1 had the highest binding capacity and most stable molecular conformation among them. Its binding energies to luteolin, kaempferol, and quercetin were −9.6 kcal/mol, −9.3 kcal/mol, and −9.2 kcal/mol, respectively (Figure 10B).

## 4 Discussion

In clinical practice, neuraminidase inhibitors are typically used to treat influenza, which is an acute respiratory illness caused by the influenza virus. Traditional Chinese medicines have long been used to treat influenza, and such medicines have several benefits compared with chemical medications, including fewer side effects, a diversity of treatments, and low drug resistance (Xi et al., 2020). YHPGs are made up of Lonicerae Japonicae Flos, Ephedra Herba, *Amygdalus Communis* Vas, Polygoni Cuspidati Rhizoma Et Radix, Licorice, and Radix Puerariae. Preliminary research has shown that these granules have several beneficial effects, including anti-inflammatory and antitussive effects, as well as the ability to prevent replication of the influenza virus. According to the concept of the gut–lung axis, intestinal and lung diseases are linked; that is, intestinal changes can lead to pathological changes in the lungs, and lung diseases can lead to intestinal damage (Budden et al., 2017; Barcik et al., 2020). Furthermore, it has been proposed that bacteria, metabolites, and free immune cells operate as dynamic mediators of the interface between the lung and the intestine (Shukla et al., 2017). Along with modulating gastrointestinal immunity, gut bacteria and their metabolites also have an impact on lung immunity through the lymphatic and blood circulations (Chellappan et al., 2019). The present study discovered that the anti-viral action of YHPGs may be directly related to the intestinal flora.

In this study, nasal drop modeling was used to induce IAV infection in mice. After successful modeling, the mice were administered YHPGs by gavage, and pathological changes in the lungs and intestinal tract were monitored to detect changes in the composition and structure of the intestinal flora, as well as in the expression of the tight junction proteins claudin-1, occludin, and ZO-1 in the colon tissues. It was discovered that mice with influenza experienced decreased eating, weight loss, and depressive symptoms. Pro-inflammatory cytokines are released concurrently with the influenza virus-induced invasion of lung tissue by inflammatory cells. In the center of the cytokine storm is a classic pro-inflammatory factor called tumor necrosis factor-α (TNF-α), and a crucial component of the inflammatory response is interleukin-6 (interleukin −6, IL-6) (Hirano, 2021; Pandey and Karupiah, 2022). The influenza virus infection can cause many organ lesions, even death, by upregulating the levels of TNF-α, IL-6, and other inflammatory cytokines and inducing a strong pro-inflammatory immune response (Alagarasu et al., 2021). Secretory IgA (sIgA) is the antibody with the highest proportion at mucosal sites and has a stronger antiviral neutralizing capacity (Berthold and Wormald, 2020). Transforming growth factor β (TGF-β) is a multifunctional cytokine that regulates multiple immune processes and activates NF-κB to induce inflammatory responses during IAV infection (Wang et al., 2012).

In the present study, the combination of lung and spleen indices, cytokine level and pathological changes in mice administered the three different doses of YHPGs suggested that this compound could reduce lung and intestinal inflammation caused by the IAV, as well as inhibit the production of pro-inflammatory cytokines and reducing the immune damage caused by the IAV, further protecting mice infected with the IAV.

The mechanical barrier function of the intestinal mucosa is determined by the permeability of the tight junction protein complexes of intestinal epithelial cells. A localized or systemic inflammatory response may occur if the intestinal barrier is compromised and permeability is increased (Bhattacharyya et al., 2014; Montilla et al., 2014). SCFAs are a byproduct of fermentation produced by intestinal bacteria that feed colonic epithelial cells and promote the aggregation of tight junction proteins in the intestine (Miao et al., 2016). Claudin-1, occludin, and ZO-1 are the three most important tight junction proteins. ZO-1 principally maintains the integrity of the tight junction complex by connecting claudin-1 and occludin. Occludin is crucial for the structure of tight junctions. By binding through the outer loops in a zipper way, occludin creates tight intercellular connections (Tan et al., 2000). In this study, we discovered that the expression of the tight junction proteins claudin-1, occludin, and ZO-1 was significantly decreased in the colon tissues of mice infected with the IAV, and that the expression of claudin-1, occludin, and ZO-1 was increased to varying degrees after treatment with YHPG. This finding suggests that YHPGs can ameliorate the damage to colon tissues caused by the influenza virus and that it has an obvious protective effect on the mucosal barrier.

In this study, we investigated two different aspects of how YHPGs influence the structural alterations in the intestinal flora of influenza-infected mice. The first aspect is how the composition of the species in the intestinal flora are affected. Based on Alpha, Beta, and differential species analysis at the phylum and genus levels, it was discovered that the intestinal flora of mice in the model group had a significantly lower richness and diversity, and the compositional structure of the intestinal flora was significantly different from that of the control group and the YHPG-treated group. Compared with the control group, the F/B dramatically decreased in the model group. Local inflammatory reactions can be induced or worsened by the decrease in Firmicutes (Kho and Lal, 2018; Stojanov et al., 2020). Proteobacteria has been proposed as a measure of intestinal flora disruption and a possible diagnostic indicator of illness, and it has been claimed that its augmentation can further worsen the inflammatory response (Shin et al., 2015). Overall, after IAV infection in mice, the composition of the intestinal flora became noticeably disorganized, and YHPGs were able to correct the disorderly structure of the flora.

At the genus level, eight genera were examined, and it was discovered that Lactobacillus, Coprobacillus, Akkermansia, Prevotella, Oscillospira, and Ruminococcus declined to varying degrees in mice in the model group. Lactobacillus is a member of Firmicutes. According to Matsusaki et al. (2016), *Lactobacillus plantarum* 06CC enhanced immunomodulatory function by increasing the amount of interleukin-12 and interferon-γ mRNA expression in Peyer’s patches. Coprobacillus and Prevotella are both butyric acid-producing bacteria. Prevotella has been reported to increase the recruitment and maturation of immune cells, as well as modifying the immunological response in the lungs (Zhao et al., 2021; Chen et al., 2022). Akkermansia is one of the important genera of intestinal bacteria. Boosting the expression of the tight junction proteins ZO-1 and occludin, which in turn influences the function of the intestinal barrier, reduces the flow of lipopolysaccharides in the bloodstream, and reduces the inflammatory response in the body (Li et al., 2016). Ruminococcu can also produce SCFAs, acetate, and butyrate (Vojinovic et al., 2019). Desulfovibrio can create hydrogen sulfide and has been identified as a potential opportunistic human pathogen (Verstreken et al., 2012). Research supports the use of probiotics as an adjuvant treatment for IAV H1N1 to boost the amount of Lactobacillus bacteria in the gastrointestinal system and enhance therapeutic results.

The second aspect is how YHPGs affect the metabolites of the gut flora. Acetate, butyrate, and propionate, among other SCFAs, are significant metabolites of intestinal bacteria. The most prevalent SCFAs in the human body are acetic acids, propionic acids, and butyric acids (Mao et al., 2022). It is believed that SCFAs facilitate communication between the immune system and gut microbiota. They primarily control T-cell function through histone deacetylase inhibition and G protein-coupled receptor activation (Kayama et al., 2020). Butyric acid is vital for the preservation of intestinal homeostasis and serves as a significant energy source for intestinal mucosal epithelial cells. Acetic acid is mostly produced by Bifidobacterium, while Ruminococcus, Coprobacillus, Prevotella, and Coprococcus produce butyric acid. For instance, the phosphate/butyrate kinase route and the butyryl coenzyme A/acetyl coenzyme A transfer are implicated in butyrate synthesis by Coprococcu (Koh et al., 2016). By inhibiting the VRG site in PB2, it has been discovered that acetyl coenzyme A can prevent replication of the influenza virus (Hatakeyama et al., 2014). The natural immune system, which is crucial for preserving intestinal homeostasis, can be stimulated by *Clostridium butyricum* and can be inhibited from overexpressing inflammatory factors (Morishita et al., 2021; Stoeva et al., 2021).

## 5 Conclusion

In this study, we discovered that YHPGs significantly reduce the onset of lung and intestinal inflammation caused by the IAV. Moreover, they inhibit the production of pro-inflammatory cytokines, decrease immune damage caused by influenza viruses, improve colonic tissue damage caused by influenza viruses, and have a significant protective effect on the mucosal barrier. This may be achieved by modulating the gut microbiota and upregulating the levels of short-chain fatty acids in the gut. YHPGs have anti-influenza virus effects, and the current study shows that one of the mechanisms underlying these effects may be their regulatory effects on the composition and structure of intestinal microbes and the release of microbial metabolites.

## Data availability statement

The datasets presented in this study can be found in online repositories. The name of the repository and accession number can be found below: National Center for Biotechnology Information (NCBI)BioProject, https://www.ncbi.nlm.nih.gov/bioproject/, PRJNA1096645.

## Ethics statement

The study was conducted in accordance with the guidelines of the Animal Ethics Committee of Zhejiang Province and the ethical number is 2023R0014. The study was conducted in accordance with the local legislation and institutional requirements.

## Author contributions

CY: Conceptualization, Data curation, Formal analysis, Investigation, Methodology, Validation, Writing – original draft, Writing – review & editing. JC: Conceptualization, Formal analysis, Project administration, Supervision, Writing – review & editing. HZ: Validation, Visualization, Writing – review & editing. DZ: Validation, Visualization, Writing – review & editing. HW: Supervision, Validation, Writing – review & editing. JY: Conceptualization, Funding acquisition, Resources, Software, Supervision, Writing – review & editing.
